# Ward Teaching

**Published:** 1908-01

**Authors:** 


### EDITORIAL NOTES AND COMMENTS.

The Business Office of the JOURNAL-RECORD is 1 1-2 to 5 1-2 South Broad Street.

The Editorial Office is 318-319-320 The Prudential.

Address all Business Commucications to Dr. George Brown, Manager.

Make remittances payable to THE JOURNAL-RECORD OF MEDICINE.

On matters pertaining to the Editorial and Original communications, address Dr. Bernard Wolff, Atlanta.

Reprints of original articles will be furnished at cost price. Requests for the same should always be made on THE MANUSCRIPT.

We will present, postpaid, on request, to each contributor of an original article, twenty (20) marked copies of THE JOURNAL-RECORD OF MEDICINE containing such article

### WARD TEACHING.

It is difficult to speak of this subject without treading on the toes of some who have been concerned in the heated discussion which has for some weeks past been so actively waged in this city. But the toes must move away or get used to it, for it is too important a subject to be overlooked.

To the mind of the average doctor, ward teaching, like many other things, is good and is bad at the same time. A little of it is good for the medical student and not bad for the patient, while too much is bad for both. The telling picture of a herd of medical students hustling and bustling through the wards like a bunch of mavericks, laughing incontinently at the patient's discomfiture, or shoving each other for a stolen glance at those maiden treasures, unmasked only to the moon in some gynecological subject, the feeble victim cowering in shrinking horror from the desecrating touch, is set for the play of Virtue Victorious and Villainy Vanquished, appealing powerfully to and meeting vociferous response from the gallery. In the roaring days when students walked the hospitals, Salpetriere, Hotel Dieu, St. Bart's or the Royal Infirmary, these delicate scenes may have been enacted. It is even related that a French surgeon, desperately pressed by surging students and on the point of being pushed over the patient's bed, cleared a space for himself by scooping up handfuls of blood and offering violently to throw it in the face of the aggressors. It was then a case of *suave qui peut.* The little man was pushed unceremoniously to the periphery of the crowd and there retaliated by hustling the taller and more fortunate onlookers and creating all the disorder and distraction possible. There was no grand stand in those days, all were bleacheries and the studied politesse of the rooter prevailed. If there is anything left of this custom, it is certainly not to be seen in the hospitals of Christendom, but perhaps may linger as a figment of the imagination borrowed from the reality of a boisterous past. It is in the same family group with Tennyson's "In the Hospital" with the red-headed demon maltreating the dying child and spurning the faithful dog, "drenched in the hellish oorali, that ever such things could be." Fine food for the antivivisectionist and the sensationalist-for-cause. If any doctor now living has witnessed such scenes, he would require the reputation of a Sir Galahad to sustain his tale. The notorious fact is that ward teaching, as carried out now-a-days, is a very decorous and rather tame affair. A few advanced students stand by and give respectful attention to the exposition of the case. There is no disorder, sparring for position or prurient curiosity. It is as quiet as a Dorcas Society meeting and fully as proper in spirit. The recollection of a case impressively presented to our minds has helped many of us in a difficult situation, and has, at the same time, brought that relief for which all of us strive. If there be legitimate objection to ward teaching, bring it out and it will be found that *veritas magna est et prevalebit.* In the meantime it is up to some one to pour a highflash point oil on the troubled waters.

